# *In vivo* dosimetry for testicular and scalp shielding in total skin electron therapy using a radiophotoluminescence glass dosimeter

**DOI:** 10.1093/jrr/rrab100

**Published:** 2021-10-29

**Authors:** Hiroyuki Okamoto, Kae Okuma, Hiroki Nakayama, Satoshi Nakamura, Kotaro Iijima, Takahito Chiba, Mihiro Takemori, Kyohei Fujii, Shohei Mikasa, Tetsu Nakaichi, Ako Aikawa, Shouichi Katsuta, Hiroshi Igaki

**Keywords:** in vivo dosimetry, radiophotoluminescence glass dosimeter (RPLD), total skin electron therapy (TSET), mycosis fungoides (MF)

## Abstract

Mycosis fungoides (MF) is a common, low-grade non-Hodgkin’s lymphoma of skin-homing T lymphocytes that can be treated via skin-directed radiotherapy. Our institution has implemented total skin electron therapy (TSET) with a 4.3 m source-to-surface distance (SSD) and 6 MeV electron beams with a beam spoiler. A 35-year-old male undergoing TSET desired to avoid radiotherapy-induced hair loss and temporary infertility; therefore, leakage dose to scalp and testicles was reduced with a special radiation shield composed of stacked lead sheets. The shields for the scalp and scrotal were of 3 mm and 6 mm, respectively. To assess leakage doses, a radiophotoluminescence glass dosimeter (RPLD) was placed at every fraction. The difference dose between the measured and prescribed dose at the calibration point was 2%. The top of the head and scrotal surface exhibited 18 cGy and 10 cGy, respectively. Thus, the dose to the scrotal surface was not beyond the testicular tolerance dose of 20 cGy. Results of semen analysis two months postradiotherapy were normal. There was no hair loss during or after radiation therapy. Therefore, the RPLD is a useful *in vivo* dosimeter that provides technical information on radiation shielding to allow for completion of TSET without hair loss or temporary infertility.

## INTRODUCTION

Mycosis fungoides (MF) is a common, low-grade non-Hodgkin’s lymphoma of skin-homing T lymphocytes. Radiotherapy can be directed to a specific skin area or the entire skin (total skin electron therapy [TSET]), which, when performed with the Stanford technique, provides standard irradiation to the entire skin [1, 2]. The standard dose of TSET is 30–36 Gy over 9–10 weeks. Common acute toxicities in TSET involve erythema/desquamation, blisters, hyperpigmentation, skin pain, and skin infection, which should be carefully managed. Low doses have been proposed with the aim to reduce acute toxicity [3].

The *in vivo* dosimetry in TSET should be performed to determine the distribution of dose to the patient’s skin and to verify the delivered prescribed dose to the patient’s skin is correct [2]. TSET with *in vivo* dosimetry [4] has demonstrated that flat regions such as the chest agreed with the prescribed dose, whereas special sites such as the ankle, medial thigh and axilla exhibited an underdose. Patient position and characteristics (height, obesity index) influence variations in the measured dose [5, 6].

The typical *in vivo* dosimetry consists of a diode detector, a thermoluminescent dosimeter (TLD), optically stimulated luminescent dosimeter, or radiophotoluminescence glass dosimeter (RPLD), and a radiochromic film. *In vivo* dosimetry with a diode detector has been used widely as it provides real-time measurement and is easy to handle (real-time measurements with TLDs and film are not available). However, TLDs and film have been used in *in vivo* dosimetry for a long time, and stable measurements may be obtained without complications.

In this case report, a patient undergoing TSET wanted to prevent radiotherapy-associated hair loss and infertility. The threshold dose for temporary infertility loss is 20 cGy [7], and the threshold dose for temporary alopecia in a single fraction is 2 Gy [8]. Radiation shielding can limit leakage doses but must be monitored accurately. With the aim to complete TSET without hair loss or temporary infertility, we used an RPLD as an *in vivo* dosimeter because it provides accurate reading and linearity, with fewer variation responses, fading, or overall measurement uncertainty compared with other detectors [9, 10].

**Fig. 1:**
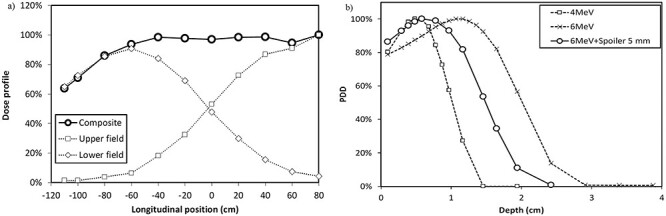
(a) The cranio–caudal dose profile for the dual-fields. (b) PDD in water equivalent phantom.

## MATERIAL AND METHODS

A 35-year-old male was first diagnosed with atopic dermatitis in 2009 and then with Stage IIB MF (T3N0M0, ISCL/EORTC [11]) in 2017. He underwent narrowband ultraviolet light B phototherapy. Treatment with oral psoralen plus ultraviolet A (PUVA) and bexarotene was continued >2 years. In 2019, facial and body skin tumor lesions emerged, which were locally irradiated by an electron beam. His doctors considered an allogeneic hematopoietic stem cell transplant, but the patient requested sperm preservation. PUVA and bexarotene were temporarily stopped, and the patient was referred to our hospital for TSET to control the lesions. The patient wanted to prevent radiotherapy-associated alopecia and fertility loss for semen cryopreservation. The radiation oncologist blocked radiation to the scalp and testicles to prevent hair loss and infertility.

Our institution implemented the Stanford University technique with 4.3 m source-to-surface distance (SSD) and 6 MeV electron beams using Varian Clinac iX (Supplementary Fig. 1). A prescribed dose of 10 Gy was administered in cycles of 2 Gy over 2 days. Fig. 1a shows a craniocaudal dose profile for dual fields of ±11° gantry angle with an acrylic beam spoiler. The patient’s soles of the feet corresponds to −120 cm (patient’s height 174 cm). The dose can be uniformly delivered to the whole body except the patient’s foot. Fig. 1b compares the percentage depth dose (PDD) with 4.3 m SSD in a water-equivalent phantom (Tough Water, Kyoto Kagaku, Kyoto, Japan) for dual fields with electron energy values of 6 MeV with a beam spoiler. All PDDs were obtained via the measured percentage depth ionization curve with a plane-parallel chamber (Roos®, PTW-Freiburg GmbH, Freiburg, Germany) [12].

Radiation shields were composed of lead with a physical density of 11.7 g/cm^3^ (Orion RadSafe Medical Co., Ltd., Tokyo, Japan). Optimum thickness was verified, and transmissions of the 3-mm- and 6-mm-thick lead sheets were 0.8% and 0.6%, respectively, as measured with an electron chamber (Roos®).

**Fig. 2:**
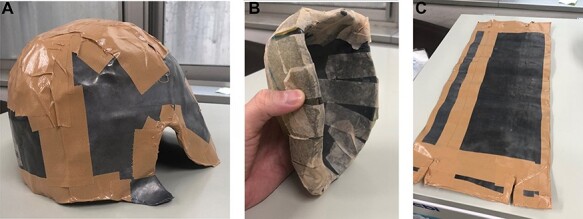
The lead shield for the head (A) and the testicles (B and C).

Fig. 2 shows the scalp helmet and bowl-shaped testicular shield were 3 mm thick. Three lead sheets were wrapped around the patient’s waist, and the lead shield for the testicles was 6 mm thick (Supplementary Fig. 2). We ensured patient comfort and ensured no gap between the radiation shield and patient’s skin at the groin for every fraction.

**Fig. 3:**
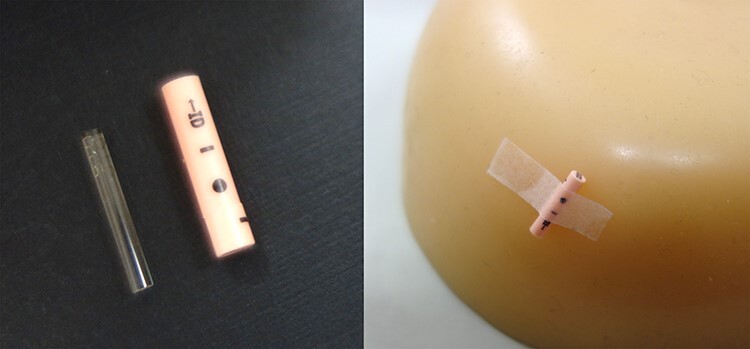
The attachment of the capsuled RPLDs to the phantom.

The RPLD dosimeter (GD-302 M, AGC Technology Solutions Co., Ltd., Kanagawa, Japan) had a 12 mm longitudinal length with 1.5 mm diameter. RPLDs were placed at a depth of 10 mm in the water-equivalent phantom and were calibrated to obtain the absorbed dose for each dosimeter. No other correction factors [13] were performed. Encapsulated RPLDs were attached by tape to the patient’s skin (Fig. 3). RPLD measurements were performed for every fraction at eight locations (Table 1 and Supplementary Fig. 2). This report was approved by the institutional ethics review board in National Cancer Center Hospital (approval number: 2017–091).

**Table 1:** The *in vivo* dosimetry using RPLDs

Cycle	#1		#2		#3		#4		#5		Sum
Number of fraction	1	2	3	4	5	6	7	8	9	10	
Top of the head (Shielding)	4	2	2	2	3	2	3	2	3	2	18
Chest (Calibration point)	*NA.*	129	63	123	76	128	66	131	66	132	915
Chest per a cycle	*NA*		186		204		197		198		785^**^
Left scrotal surface^*^	1	1	1	4	1	2	1	1	1	1	11
Right scrotal surface^*^	1	1	1	1	1	1	1	1	1	1	9
Left groin^*^	2	2	2	2	2	2	2	2	2	2	15
Right groin^*^	2	2	2	2	2	2	3	2	2	2	17
Left underside of the foot	13	11	8	10	12	9	11	12	12	9	82
Right underside of the foot	11	10	10	12	9	20	10	13	12	11	97

*NA*, Not Available; Unit of measurement is cGy;

^*^Shielding measurement points.

^**^Total dose was derived from sum of the measured dose from 2nd to last cycles, because the measurement at the 1st fraction could not be done by misplacement.

## RESULTS AND DISCUSSION

Results of *in vivo* dosimetry with RPLDs are shown in Table 1. Dosimetry for the chest at the 1st fraction could not be performed due to misplacement. Therefore, the total measured dose at the chest location was 789 cGy from the 2^nd^ cycle dose, corresponding to the delivered dose of 800 cGy. The difference between the two was within 2%. The head and scrotal surface for all fractions exhibited 18 and 10 cGy leakage doses, respectively, which were lower than the threshold [7, 8]. Semen test results according to the WHO criteria [14] two months postradiotherapy were normal (Supplementary Table 2). Hair loss was not observed.

TLDs are used most widely for *in vivo* dosimetry in TSET [5]. RPLD for *in vivo* dosimetry in TSET requires testing. To the best of our knowledge, this is the first report to examine the testicular dose through *in vivo* dosimetry using RPLD in TSET. RPLDs overcome the stem effect when measuring leakage doses [15]. In this study, the signal-to-noise ratio of the RPLDs was 500. RPLDs measurements for the chest were in good agreement with the prescribed dose and similar to *in vivo* dosimetry using TLD in TSET [5].

Supplementary Table 1 summarizes measurement uncertainty (5.2%) of RPLDs at the calibration point. For other uncertainties, angular dependency should be considered [10, 16]. In this work, placement of RPLDs at the chest was almost the same as the calibration points. However, it was impossible to place RPLDs in the same direction as the calibration points at the top of the head and scrotal surface. The effects of these remain unclear. Moreover, materials in RPLDs have a high atomic number, which might cause energy dependency and increased sensitivity of measurements as photon energy decreases [13, 17]. Energy spectra changes under radiation shielding remains unclear. However, primary radiation is likely shielded effectively under lead sheets, and bremsstrahlung radiation or scattered radiation mainly causes leakage doses. These effects lead to safe estimation of the exposure dose.

Leakage doses at the head and scrotal surface was 1.8% and 1.0%, respectively, and slightly greater than the phantom measurements (0.8% and 0.6%). That was because the shapes of the lead sheets were different to cover the scrotal and head skin surfaces and six different standing positions were required; radiation shielding might be inadequate especially for the back of the patient’s body. However, lead shielding successfully maintained leakage doses below the tolerance dose. Our work demonstrates that a RPLD is a reliable and useful *in vivo* dosimeter for TSET.

## Supplementary Material

supplementary_material_rrab100
