# Supplementary material for: In vivo dosimetry for testicular and scalp shielding in total skin electron therapy using a radiophotoluminescence glass dosimeter
Source: J Radiat Res. 2021 Oct 29;63(1):51–4. doi: 10.1093/jrr/rrab100 (PMC8776692; doi:10.1093/jrr/rrab100)
Supplement: supplementary_material_rrab100 [file supplementary_material_rrab100.pdf]

Supplementary materials

a)

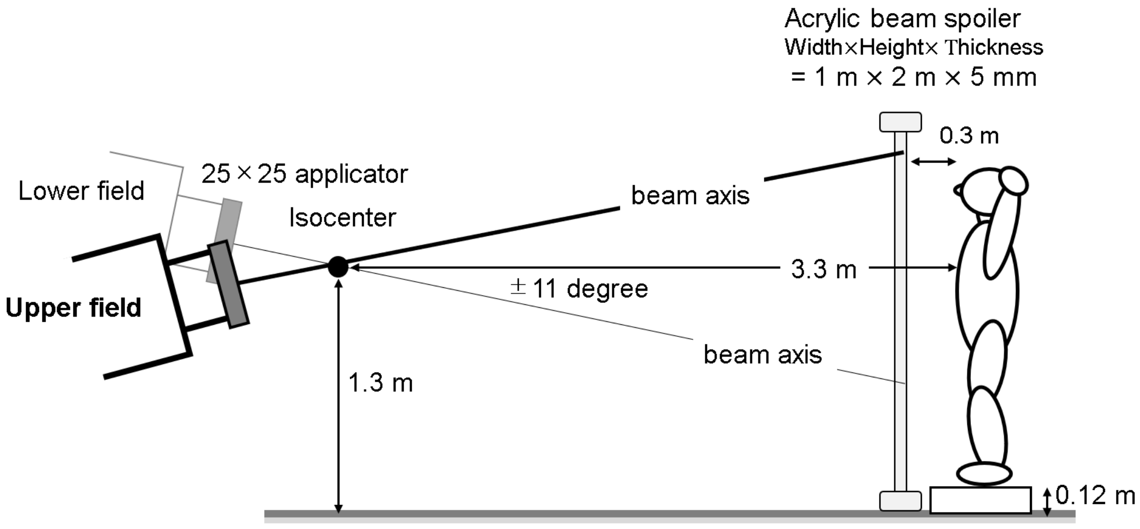

b)

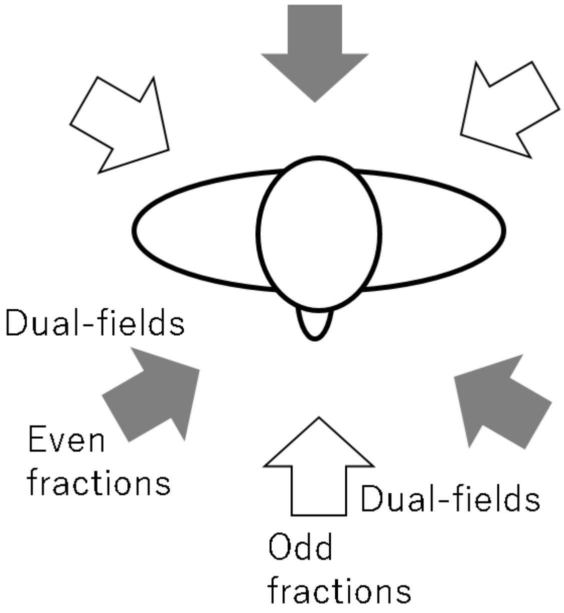

Figure 1. a) The Stanford techniques. The beam spoiler with a thickness of 5 mm was set at a distance 0.3 m from the patients. b) The six different standing positions with a cycle of 2 Gy/2days.

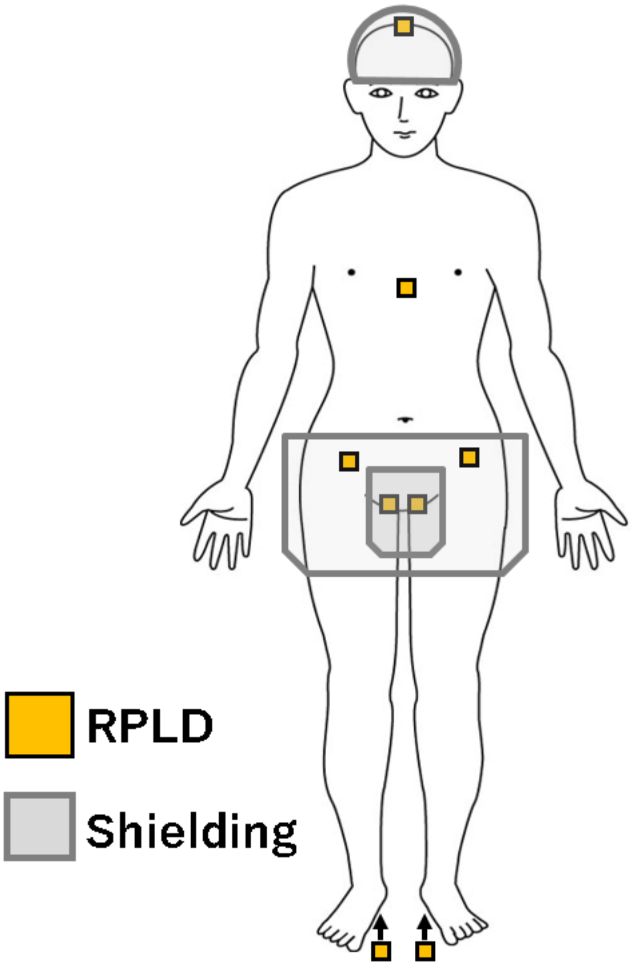

Figure 2. The schema for location of RPLDs and the lead sheets.

RPLD: a radio-photoluminescence glass dosimeter.

Table 1. The measurement uncertainty for in-vivo dosimetry

using RPLDs at the calibration point in TSET.

Description	Uncertainty
(i) Reproducibility of reading	0.2%
(ii) Individual response	2.0%
Reproducibility of reading	0.2%
Reproducibility in long term	2.0%
(iii) Calibration factor	1.7%
Absorbed dose in water	1.7%
Reproducibility of reading	0.2%
Combined uncertainty	2.6%
Expanded uncertainty U ($k = 2$)	5.2%

Table 2. Semen test results according the WHO criteria

Test	WHO criteria	Results
Semen volume	> 1.5 ml	2.7 ml
Sperm concentration	> 1.5×10^6 ml	24.5×10^6 ml
Sperm total motility	>40%	58.2%
Sperm with a normal morphology	>4%	54.5%
